# Role of Cardiac Magnetic Resonance in Detecting Biventricular Apical Hypertrophic Cardiomyopathy

**DOI:** 10.1155/2021/8833216

**Published:** 2021-02-09

**Authors:** Nathan Zaher, Hammam Shereef, Rashid Al Hussain, John Dawdy, Diane Levine, M. Chadi Alraies

**Affiliations:** ^1^Department of Internal Medicine, Wayne State University/Detroit Medical Center, Michigan, USA; ^2^Department of Internal Medicine, Beaumont Hospital-Dearborn, Michigan, USA; ^3^Division of Cardiology, Wayne State University/Detroit Medical Center, Michigan, USA

## Abstract

Apical Hypertrophic Cardiomyopathy (ApHCM) is a rare variant of hypertrophic cardiomyopathy with a low prevalence in the general population. ApHCM with right ventricular involvement (BiApHCM) is largely unreported and may not be detected with conventional transthoracic echocardiogram (TTE) alone. Cardiac Magnetic Resonance (CMR) has been demonstrated to be a proficient imaging modality to diagnose BiApHCM. We present a case of BiApHCM that was diagnosed with TTE and further characterized by CMR. This imaging modality may be utilized more in the future to help diagnose and detect the prevalence of BiApHCM.

## 1. Introduction

Apical Hypertrophic Cardiomyopathy (ApHCM) is a rare variant of hypertrophic cardiomyopathy involving the left ventricle. ApHCM with right ventricular (RV) involvement (BiApHCM) is largely unreported and may not be detected with transthoracic echocardiogram (TTE). Cardiac Magnetic Resonance Imaging (C-MRI) has superior sensitivity and provides additional important details related to cardiac thickness and function. We herein present a case of BiApHCM that was diagnosed with TTE and further characterized by C-MRI. This imaging modality provides superior quality and may be considered for screening purposes in appropriately selected patients with clinically suggestive findings and negative echocardiograms. It has also been shown to be a better determinate of LV wall thickness which has a significant impact on management decisions and patient outcomes.

## 2. Case Description

**Figure 1:**
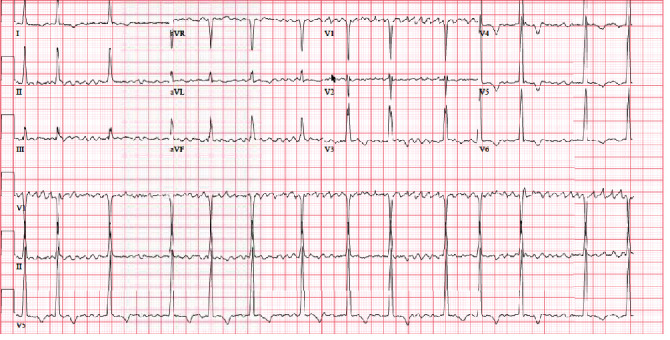
12-lead EKG showed absence of *P* wave, baseline fibrillary waves, increased LVH by Sokolow-Lyon criteria, and RSR pattern in V2 and V3.

**Figure 2:**
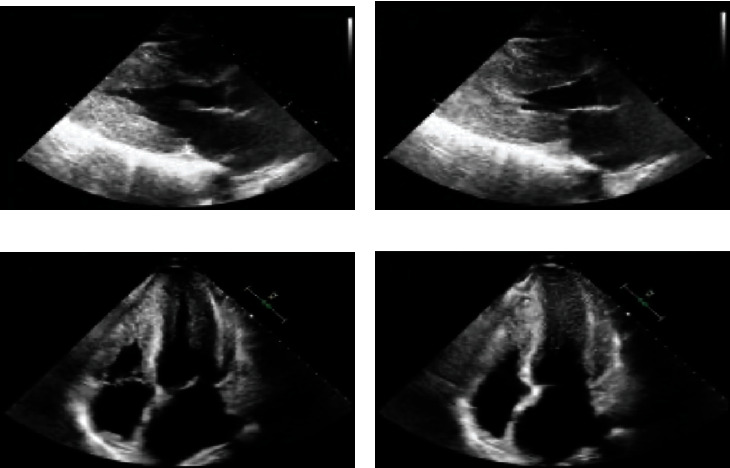
2D echocardiography parasternal long-axis view in diastole (a) and systole (b) along with apical 4-chamber view in diastole (c) and systole (d) showing apical hypertrophy in both left and right ventricles (first attachment).

**Figure 3:**
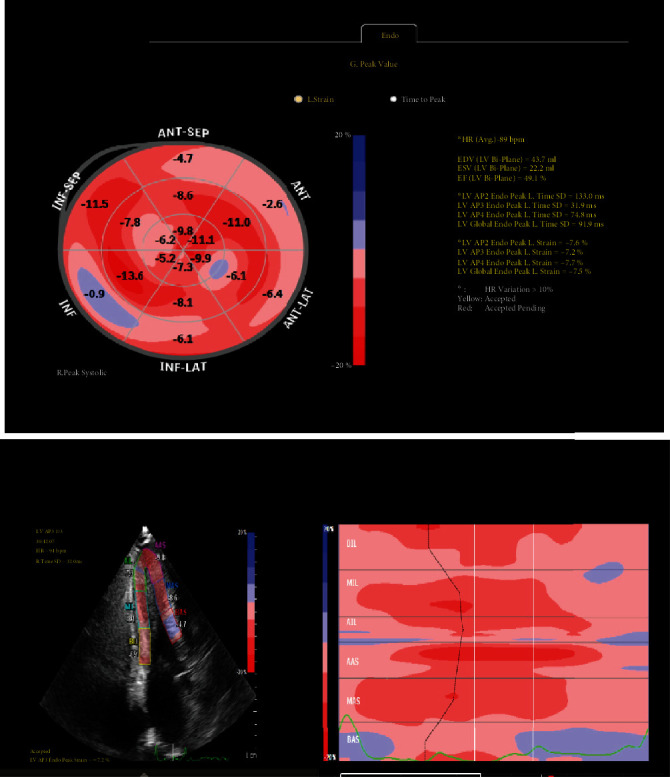
The longitudinal strain bull's eye plot showing concentric hypertrophy and normal EF is characterized by a mildly reduced average global and prominently reduced longitudinal strain of multiple nine segments.

**Figure 4:**
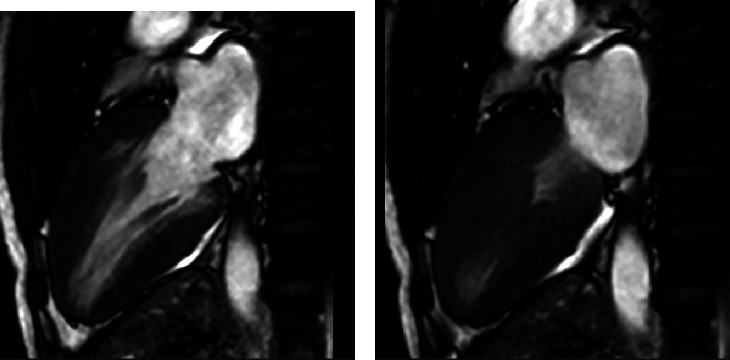
(a) Severe concentric left ventricular hypertrophy with focal maximum thickness of myocardium 20 mm in the mid anterior wall. (b) Globally hyperdynamic left ventricular systolic function with calculated LV EF 80% and near obliteration of mid and distal segments in systole.

**Figure 5:**
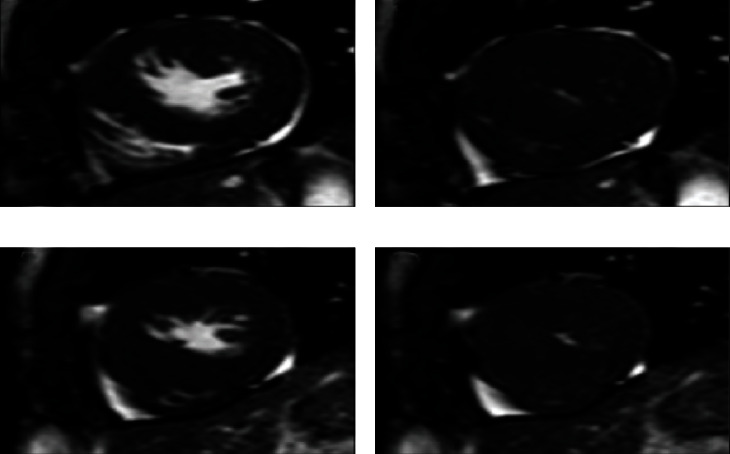
MRI short-axis view showing mid-LV chamber at the end of diastole (a) with obliteration of cavity in end systole (b). MRI short-axis view showing apex in end diastole (c) with obliteration of cavity in systole (d). Hypertrophy can be appreciated in both the left and right ventricles.

**Figure 6:**
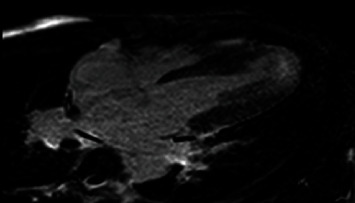
A 4-chamber long axis inversion recovery showing late gadolinium enhancement in the apex.

A 55-year-old man presented to the Emergency Department (ED) with palpitations, shortness of breath, and nonexertional pleuritic chest pain and was found to be in atrial fibrillation. His vitals were stable, and there was a systolic grade 3/6 murmur loudest at the left sternal border on physical exam. A 12-lead EKG was obtained and revealed underlying atrial fibrillation rhythm with increased voltage criteria of left ventricular hypertrophy and right bundle branch block with RSR pattern in V2 and V4 (Figure 1). A TTE was ordered and demonstrated hyperdynamic systolic function and exuberant apical hypertrophy with right ventricular involvement (Figure 2). Offline longitudinal strain analysis was performed at appropriate frame rates and was consistent with BiApHCM (Figure 3). A C-MRI, without and with contrast, demonstrated nonobstructive cardiomyopathy with RV thickening (Figures 4–6). The thickening was pronounced in the midapical area with a wall measurement of 6.5 + 1.3 mm at the midapical segment. The patient was managed with metoprolol both for rate control of atrial fibrillation and to maximize diastolic filling time and cardiac output. He was instructed to follow-up as an outpatient for genotyping/cascade screening.

## 3. Discussion

Apical Hypertrophic Cardiomyopathy (ApHCM), a rare form of HCM, manifests as hypertrophy of the left ventricular apex and has nonobstructive physiology. Research has found sarcomere mutations can cause apical hypertrophy, such as the cardiac actin Glu101Lys [1]. The apical variant is most commonly seen in the Japanese population with a report of 15-25% prevalence in documented case series of HCM, in comparison to only 3% of patients with HCM in the general population [2]. Diagnostic studies include left ventriculography, contrast transthoracic echocardiogram (TTE), Computed-Tomography (CT), and Cardiovascular Magnetic Resonance (CMR) [3].

Obstruction in BiApHCM is due to midsystolic contact of the prominent RV muscle bundles in the RV outflow tract. Patient presentation and approach to treatment in BiApHCM are similar to that of left-sided ApHCM. First-line therapy is an AV nodal blocking agent. Those who remain symptomatic can be offered resection of accessory RV muscles in the outflow tract [4, 5]. Despite being the most sensitive and accurate imaging modality for BiApHCM, C-MRI has not been widely obtained due to cost-related limitations [6]. One study involving 330 myocardial segments showed that thickness could be measured by echocardiography in 221 (67%) vs. 320 (97%) with C-MRI. Reliance on routine echocardiography as the sole imaging modality to rule out ApHCM has led to underreporting of this condition with potentially suboptimal outcomes [7]. This is also of important clinical significance as patients with massive LVH (>30 mm) are often considered high risk and should be considered for ICD therapy [8].
